# The *Arabidopsis thaliana *response regulator ARR22 is a putative AHP phospho-histidine phosphatase expressed in the chalaza of developing seeds

**DOI:** 10.1186/1471-2229-8-77

**Published:** 2008-07-15

**Authors:** Jakub Horák, Christopher Grefen, Kenneth W Berendzen, Achim Hahn, York-Dieter Stierhof, Bettina Stadelhofer, Mark Stahl, Csaba Koncz, Klaus Harter

**Affiliations:** 1 Zentrum für Molekularbiologie der Pflanzen/Pflanzenphysiologie, Universität Tübingen, Auf der Morgenstelle 1, D-72076 Tübingen, Germany; 2 Zentrum für Molekularbiologie der Pflanzen/Mikroskopie, Universität Tübingen, Auf der Morgenstelle 3, D-72076 Tübingen, Germany; 3 Max Planck Institut für Züchtungsforschung, Carl-von-Linné-Weg 10, D-59829 Köln, Germany

## Abstract

**Background:**

The *Arabidopsis *response regulator 22 (ARR22) is one of two members of a recently defined novel group of two-component system (TCS) elements. TCSs are stimulus perception and response modules of prokaryotic origin, which signal by a His-to-Asp phosphorelay mechanism. In plants, TCS regulators are involved in hormone response pathways, such as those for cytokinin and ethylene. While the functions of the other TCS elements in *Arabidopsis*, such as histidine kinases (AHKs), histidine-containing phosphotransfer proteins (AHPs) and A-type and B-type ARRs are becoming evident, the role of ARR22 is poorly understood.

**Results:**

We present evidence that ARR22 is a preferentially cytoplasmic protein, exclusively expressed in the chalaza of developing seeds. ARR22 specifically interacts with AHP2, AHP3 and AHP5 in yeast and living plant cells. Two new loss-of-function alleles, *arr22-2 *and *arr22-3*, were isolated and characterized. With respect to their morphology and metabolite status, no significant difference in the developing seeds of the *arr22 *mutants was observed compared to wild type. The genetic complementation of the *arr22 *mutants with a genomic *ARR22 *fragment resulted in plants (*arr22*/*gARR22*) with a pleiotropic phenotype of different penetrance. This phenotype was not observed when the phosphorylatable Asp74 of ARR22 was changed to either a dominant-active Glu or a dominant-inactive Asn. The phenotype of the *arr22*/*gARR22 *plants was comparable to that of multiple *ahk*, *ahp *and *B-type arr *mutants.

**Conclusion:**

Our results favor the model that ARR22 acts as a phospho-histidine phosphatase on specific AHPs in the cytoplasm of *Arabidopsis *chalaza cells. The lack of any aberrant morphological and metabolite phenotype in the seeds of the *arr22 *mutants indicates that ARR22 is probably primarily responsible for the fine tuning of specific branches of chalaza-based TCS signalling. Even when slightly mis-expressed, ARR22 interferes with hormone homeostasis in non-chalaza tissues. Our data indicate that the chromatin status might play a crucial role in maintaining the chalaza-restricted expression of *ARR22*.

## Background

Two-component systems (TCSs) have emerged as important signal response mechanisms in higher plants [1-4]. TCSs were originally described in prokaryotic systems, where they perceive and process a wide range of environmental signals [5,6]. In plants, canonical TCSs play major roles in mediating physiological responses to hormones, such as cytokinin and ethylene [7-10], nutrients [11,12] and light [13-15] and are also important for the ethylene and H_2_O_2 _triggered stomatal closure response [16,17]. Furthermore, TCS elements maintain the pace of the circadian clock and mediate the input of the cytokinin signal to its circadian phase [18,19]. TCS components have also been shown to be important for the regulation of several developmental processes, such as the control of the number of stem cells in the shoot apical meristem [20,21], the development of the female gametophyte [22,23], the differentiation of root vascular tissue [24-26] and the formation of root nodules during *Rhizobium*/plant interaction [27,28].

Analysis of the genome of *Arabidopsis thaliana *and other plants has revealed sequences encoding proteins that are homologues to all three elements required to establish the complex type of two-component system and a His-to-Asp phosphorelay: the hybrid histidine kinases (AHKs), the histidine-containing phosphotransfer proteins (AHPs) and the response regulators (ARRs). The ARRs are further subdivided into three discrete groups, the A-type ARRs and B-type ARRs [29] and a novel group of ARRs consisting of ARR22 and ARR24. The receiver domains of ARR22 and ARR24 have higher similarity to the receiver domains of hybrid histidine kinases than to those of the other response regulators [30].

The capacity of TCS proteins to act within a phosphorelay, as histidine kinases, phosphotransfer proteins, response regulators or phospho-histidine phosphatases has been demonstrated for representative members of each group [3,30]. Most of the present data can be combined to an attractive, general model of TCS signalling as it is exemplarily proposed for the cytokinin response pathway [2,4]: Upon activation by its appropriate stimulus the AHK undergoes autophosphorylation at a conserved His residue in its transmitter domain. The phosphoryl residue is then transferred to a His in the AHPs *via *an Asp in the receiver domain of the AHK. The AHPs distribute the phosphoryl residue to either nucleo-cytoplasmic A-type ARRs, nuclear B-type ARRs or, as exemplarily demonstrated for AHP5, can be dephosphorylated by ARR22 in vitro.

In *Arabidopsis*, the canonical TCS elements are encoded by multigene families. Whereas the AHP family consists of 6 members, the A-type and B-type ARRs subfamilies are each represented by 11 members. The B-type ARRs are transcription factors acting as partially redundant positive regulators of cytokinin signal transduction by modulating the expression of cytokinin response genes, including *type-A ARRs *[9,31]. Recently, Hutchinson and colleagues [7] reported that AHPs also function as partially redundant positive regulators of cytokinin signalling in *Arabidopsis*. The A-type ARRs, however, are proposed to be partially redundant negative regulators of cytokinin signalling [15] but seem to have a more general function in the integration and coordination of several signalling processes, such as those of light [13,14], the circadian clock [18,19] and control of stem cell number [20,21].

Despite their generally redundant function in cytokinin signalling, some members of the A-type and B-type ARRs show a highly specific expression pattern. For instance, the B-type response regulators *ARR18 *and *ARR20 *are expressed in developing anthers and in the tip of the pistil, respectively [32]. Comparably, the expression of the response regulators *ARR22 *and *ARR24*, which form a paralogous gene pair in *Arabidopsis*, is proposed to be restricted to the funiculus/chalaza junction in the developing seed and pollen grains, respectively [33]. Furthermore, ARR22 functions as a phospho-histidine phosphatase on phosphorylated AHP5 *in vitro *[30]. Despite this activity and specific expression, loss-of-function alleles of either *ARR22 *or *ARR24*, displaying an aberrant but specific phenotype, could not be identified so far [33]. However, as reported by Kiba and colleagues [30], the ectopic expression of *ARR22 *in *Arabidopsis *induced a dwarf phenotype with a poorly developed root system and a de-regulation of cytokinin-responsive genes. This suggests that ARR22, when mis-expressed, interferes with the cytokinin response pathway in *Arabidopsis *[30].

In this study we show that ARR22 is a predominantly cytoplasmic protein, whose expression is restricted to the chalaza of developing seeds. Surprisingly, the analysis of two novel *arr22 *loss-of-function alleles, with respect to seed development, nutrient content and composition, revealed no abnormalities. The interaction of ARR22 with a subset of AHPs and the different abnormal developmental phenotypes of transgenic *arr22 *plants complemented with a genomic *ARR22 *fragment, however, suggest that even small traces of ARR22, expressed in non-chalaza tissues, greatly disturb the plants' TCS signalling network.

## Results

### *ARR22 *is specifically expressed in the chalaza of developing seeds

**Figure 1:**
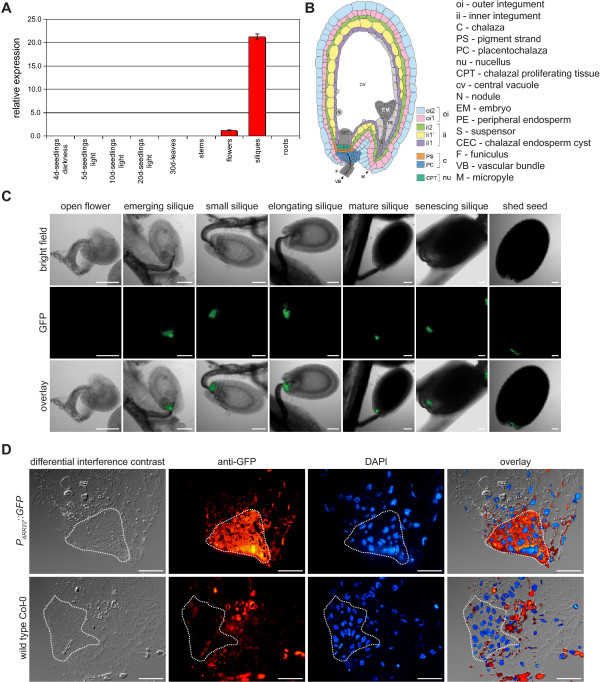
**ARR22 is expressed in the chalaza of developing seeds**. (A) Steady-state *ARR22 *transcript levels in different organs and at different developmental stages of *Arabidopsis *as determined by quantitative RT-PCR. Data are expressed as mean +/- SD (n = 3) (B) Scheme of *Arabidopsis *seed anatomy adapted according to Debeaujon and colleagues [62]. Note the location of the chalaza. (C) Confocal images of transgenic seeds at different developmental stages expressing a *P*_*ARR22*_*::GFP *construct. Bright field, GFP images and overlays are presented from the left to the right. The bars represent 100 μm. (D) Immunolocalization of GFP on cryosections prepared from developing seeds of *P*_*ARR22*_*::GFP *transgenic plants using a GFP-specific antibody (anti-GFP). The nuclei of the cells were visualised by DAPI staining (DAPI). The dotted line confines the chalaza area. The bars represent 20 μm.

To define the spatial and temporal expression of *ARR22 *in *Arabidopsis*, we first performed quantitative RT-PCRs using RNA from different tissues and developmental stages. The data demonstrated that the *ARR22 *transcript was only detectable in flowers and more predominantly in siliques (Fig. 1A). Subsequently, we translationally fused the *ARR22 *promoter to the GFP gene. The used 2003 bp long *ARR22 *promoter fragment covered the entire sequence 1608 bp upstream of the transcription start and 395 bp of the 5' UTR and was expected to contain all significant regulatory elements [34,35]. Confocal laser scanning microscopy (CLSM) of transgenic *Arabidopsis *plants carrying this reporter construct indicated that GFP fluorescence was restricted to the chalaza cells of developing seeds (Fig. 1B, C). The activity of the *ARR22 *promoter was first detectable in the seeds shortly after fertilization (emerging silique) and persisted through all the following developmental stages until the seed was shed. We also fused the *ARR22 *promoter fragment to the *uidA *reporter gene and generated *P*_*ARR22*_*::uidA*-containing transgenic plants. The GUS staining slightly extended to non-chalaza cells in the developing seeds. However, we could not detect specific GUS activity in organs other than the developing seeds (data not shown; [33]). To confirm the specific chalazal expression of *ARR22*, we performed fluorescence immunolabelling on 300–500 nm cryosections of seeds from elongating siliques (Fig. 1C). The GFP protein was only detected in the chalaza indicating the spatially and temporally restricted expression of *ARR22 *in *Arabidopsis *(Fig. 1D). The observed *P*_*ARR22 *_activity pattern is supported by recent microarray data which demonstrate the accumulation of *ARR22 *transcript solely in developing seeds [36].

Using the *P*_*ARR22*_*::GFP *reporter lines, we also tested the activity of the *ARR22 *promoter in the chalaza in response to cytokinin and ethylene. For this purpose, we soaked excised inflorescences in either water or solutions containing different concentrations of cytokinin (benzyladenine) or the ethylene precursor 1-aminocyclopropane-1-carboxylic acid. At different time points after hormone application we excised the siliques and analysed the chalaza for its fluorescence intensity. However, no induction of the *ARR22 *promoter by cytokinin or ethylene was observed (data not shown).

### ARR22 is predominantly localized in the cytoplasm of plant cells

**Figure 2:**
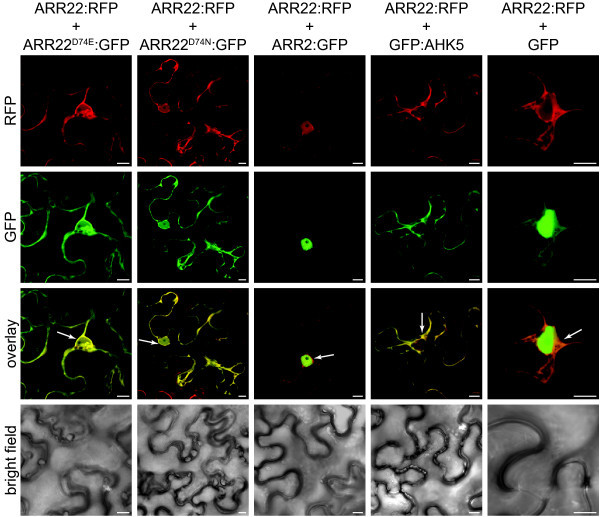
**ARR22 is predominantly localized in the cytoplasm of tobacco leaf cells**. Confocal images of abaxial epidermal leaf cells expressing the indicated RFP and GFP fusion proteins are shown. The emission channels for the RFP (ARR22:RFP) and GFP (marker fusion proteins) fluorescence and the overlay are indicated at the left. The lowest row shows the bright field images of the transformed cells. Marker genes are ARR2 for the nucleus, AHK5 for the cytoplasm and plasmalemma and GFP for nucleo-cytoplasmic distribution. The white arrows indicate the dense cytoplasm around the nucleus. The bars represent 10 μm.

We next examined the subcellular localization of wild type ARR22 and point-mutated versions in which the conserved phosphorylatable aspartate (D74) was changed to either non-phosphorylatable glutamate (E) or asparagine (N). For this purpose, we expressed wild type ARR22 fused to RFP and the modified ARR22^D74E ^and ARR22^D74N ^proteins fused to GFP, under the control of the constitutive Cauliflower Mosaic Virus (CaMV) 35S promoter, transiently in tobacco (*Nicotiana benthamiana*) leaf cells. For the verification and identification of the nuclear and cytoplasmic compartments we used B-type ARR2:GFP (nucleus; [37]), AHK5:GFP (cytoplasm and plasmalemma; [38]) and GFP (cytoplasm and nucleus) as markers. The leaves were co-infiltrated with *Agrobacterium *strains carrying either the RFP or GFP constructs and a strain conferring the expression of the silencing inhibitor gene *p19 *[39]. After transformation, the abaxial epidermis of the tobacco leaves was subjected to CLSM. We observed a strong RFP fluorescence in the cytoplasm and a weaker signal inside the nucleus indicating that ARR22 is predominantly a cytosolic protein as compared to the nuclear marker ARR2:GFP protein (Fig. 2, RFP). This subcellular localization of ARR22 was not affected by the exchange of Asp74 to Glu or Asn (Fig. 2).

### ARR22 interacts with a subset of phosphotransfer proteins in yeast

**Figure 3:**
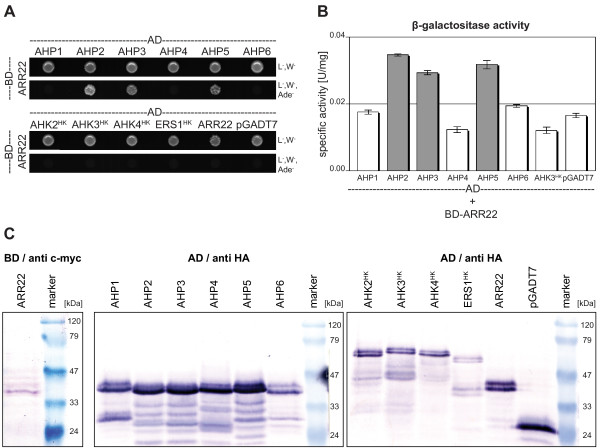
**ARR22 interacts with a subset of AHPs in the yeast two-hybrid assay**. (A) Growth assay. Yeast clones expressing BD-ARR22 and the indicated AD clones were cultivated for 4 days at 28°C on either vector selective media (L^-^, W^-^) or interaction selective media (L^-^, W^-^, Ade^-^). The empty pGADT7 vector expressing the AD domain only was used as a negative control. (B) Quantitative β-galactosidase (*LacZ *reporter gene) activity assay. The specific β-galactosidase activity was measured in the extracts of three independent yeast clones expressing BD-ARR22 and the indicated AD fusion protein. Background activity (white bars) was determined for extracts from clones not growing on interaction selective media. Extracts from yeast clones showing above-background activity and demonstrating *in vivo *interaction are indicated in grey. Data are expressed as mean +/- SD (n = 3). (C) Western-blot detection of the fusion proteins expressed in the yeast cells used for the two-hybrid analysis. The immunodetection of the BD-ARR22 was carried out with an antibody directed against the c-myc tag (anti c-myc). The AD fusion proteins were detected with an antibody directed against the HA tag (anti HA). 15 μg of total protein were loaded per lane.

To identify TCS partners of ARR22 potentially involved in a chalaza-based phosphorelay, we screened for protein interactions in the yeast two-hybrid system. We cloned the *ARR22 *cDNA in the "bait" vector pGBKT7-DEST and the cDNAs of all *AHPs *(*AHP1 *to *AHP6*) as well as the cDNA fragments encoding the histidine kinase (HK) domain of the cytokinin receptors (*AHK2 *to *AHK4*) and the ethylene receptor *ERS1 *in the "prey" vector pGADT7-DEST. The HK domain of the AHKs and ERS1 was used to address the question whether ARR22, which is more similar to the receiver domains of hybrid His kinases than to the receiver region of response regulators [30], could interfere with either the hybrid His kinases or with ERS1, the only plant non-hybrid His kinase. The *BD-ARR22 *construct was first transformed into the yeast strain PJ69-4A and then a BD-ARR22 expressing clone was re-transfected with the prey *AD *constructs shown in Figure 3. The interaction of the proteins was assessed by growth assay on interaction-selective media and measuring *LacZ *reporter gene activity (β-galactosidase) (Fig. 3A, B). Although all AD fusion proteins were expressed (Fig. 3C), only the yeast clones containing the *AHP2*, *AHP3 *or *AHP5 *construct together with *BD-ARR22 *grew on interaction selective medium and displayed β-galactosidase activity above the background level (Fig. 3A, B). No interaction of ARR22 was found with AHP1, AHP4 and AHP6 and the HK domains of the histidine kinases (Fig. 3). Furthermore, ARR22 did not form homodimers in yeast (Fig. 3).

### ARR22 interacts with AHP2, AHP3 and AHP5 in plant cells

**Figure 4:**
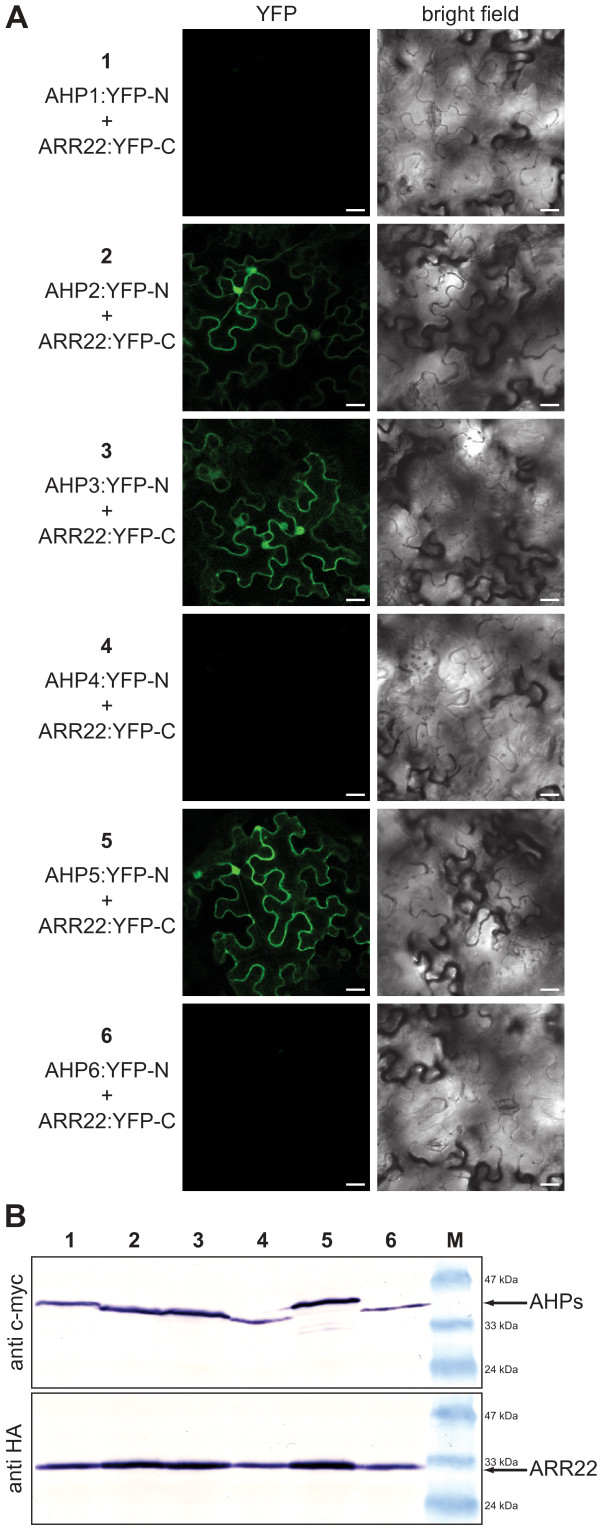
**ARR22 specifically interacts with AHP2, 3 and 5 in tobacco leaf cells**. (A) Confocal images of abaxial epidermal tobacco leaf cells expressing the indicated YFP-N and YFP-C fusion proteins (left column). The right column shows the corresponding bright field images of the transformed cells. The bars represent 25 μm. (B) Western blot analysis of protein extracts derived from transiently transformed tobacco leaves assayed for BiFC fluorescence before extraction (1–6). Immunodetection of the YFP-N fusion proteins (AHPs) was carried out with an antibody against c-myc-tag (anti c-myc) and of the YFP-C fusion protein (ARR22) with an antibody against the HA-tag (anti HA). M, protein marker.

To substantiate the ARR22 interactions observed in yeast, we studied protein-protein interactions in plant cells using bimolecular fluorescence complementation (BiFC; [40]). For this purpose, we generated 35S promoter-driven BiFC constructs expressing ARR22 in fusion with the C-terminal YFP fragment (YFP-C) and the AHP fusions with the N-terminal YFP fragment (YFP-N; Fig. 4). Western-blot analysis confirmed that the fusion proteins were co-expressed in *N. benthamiana *leaf cells in the presence of the p19 silencing inhibitor (Fig. 4B). As shown in Figure 4A, a significant BiFC signal and, thus, interaction was only observed when ARR22 was co-expressed with AHP2, AHP3 and AHP5. No ARR22 interaction was observed with AHP1, AHP4 and AHP6 (Fig. 4A). Thus, in support of our yeast data, ARR22 showed specific interaction with a subset of AHPs *in planta *and not with any of the other two-component signalling elements tested.

### Isolation and characterization of *ARR22 *insertion mutants

**Figure 5:**
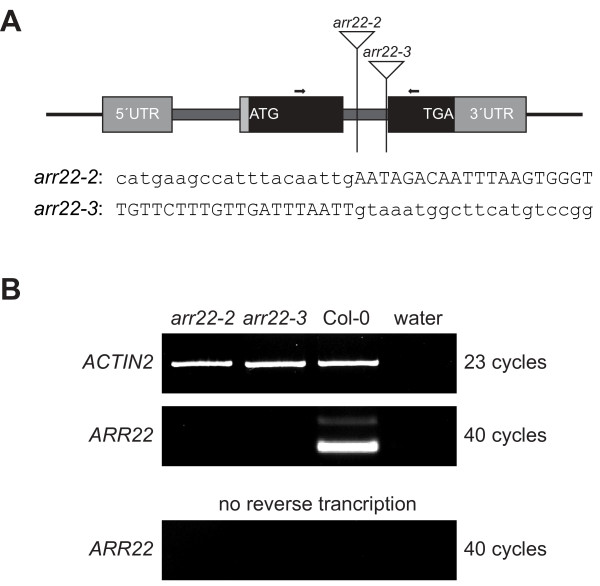
**Characterization of *ARR22 *T-DNA insertion mutants**. (A) Scheme of the *ARR22 *(At3g04280) locus and positions of T-DNA insertions. The exons are depicted as filled boxes (coding region in black, UTR in grey), introns as thick lines, and the T-DNA insertions as triangles. The sequences at the insertion of the T-DNA (lower case letters) into the *Arabidopsis *genome (upper case letters) are given for both alleles (*arr22-2*, *arr22-3*). Arrows indicate the sites of primers used for RT-PCR analysis. (B) End-point RT-PCR analysis of the steady-state level of *ARR22 *transcript. The cDNA was derived from total RNA extracted from siliques of the two allelic homozygous *arr22 *T-DNA insertion lines (*arr22-1*, *arr22-2*) and wild type (Col-0). PCR was perfomed with the *ARR22*-specific primers indicated in (a) and, as a control, with *ACTIN2*-specific primers. To exclude any cross-contamination and contamination with genomic DNA, the RT-PCR was performed in the absence of total RNA or without its reverse transcription.

For the further analysis of the *ARR22 *function, we identified two T-DNA insertion mutant alleles of the *ARR22 *gene by screening the Cologne collection, representing 90,000 independent T-DNA insertion lines in the Col-0 ecotype [41]. Both mutant lines (*arr22-2*, *arr22-3*) contained the T-DNA insertion in the second intron of the *ARR22 *gene (Fig. 5A). In the *arr22-2 *mutant the T-DNA was inserted 288 bp downstream of the start codon, whereas in the *arr22-3 *line it was located 372 bp downstream of the start ATG, corresponding to a position 3 bp upstream of the 3' splicing site of intron 2 (Fig. 5A). After the isolation of homozygous lines, RNA from siliques was tested for the presence of the *ARR22 *transcript by end-point RT-PCR (Fig. 5B). In contrast to wild type, no *ARR22 *transcript was detected in either of the insertion mutant lines. The weaker band with slower electrophoretic mobility in the wild type preparation represents an additional *ARR22 *transcript with an unspliced second intron [33]. A contamination with genomic DNA was ruled out by PCR amplification without reverse transcription which did not produce *ARR22 *amplicons. Compared to wild type, however, both mutant lines showed no aberrant phenotype with respect to their vegetative development.

### Impact of the loss of *ARR22 *function on seed development and nutrition

**Figure 6:**
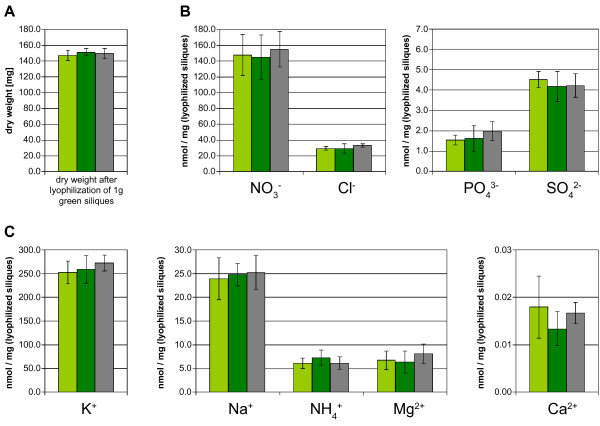
**Seeds of the *arr22 *mutants and the wild type show no difference in their dry weight and content of inorganic ions**. The dry weight (A) and the amount of the indicated inorganic ions (B) was determined in the *arr22-2 *(light green bars), *arr22-3 *(dark green bars) mutant and wild type (grey bars) by HPLC analysis as described in Methods. The data are expressed as mean ± SD (n = 5).

**Figure 7:**
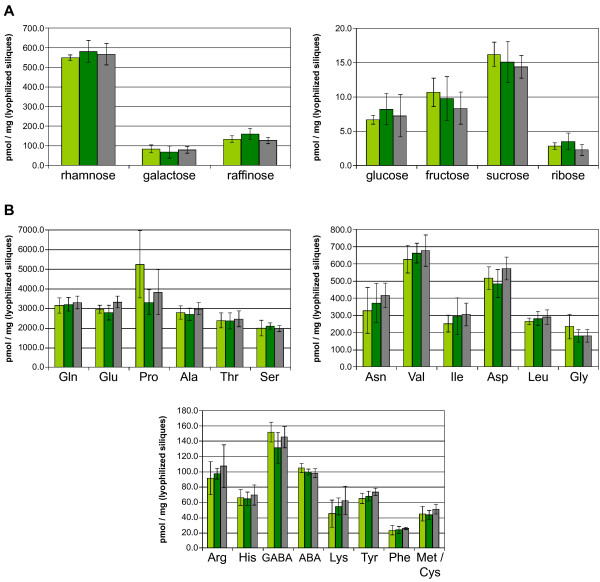
**Seeds of the *arr22 *mutants and the wild type show no difference in their carbohydrate and amino acid content**. The amount of the indicated sugars (A) and amino acids (B) was determined in the *arr22-2 *(light green bars), *arr22-3 *(dark green bars) mutant and wild type (grey bars) by HPLC analysis as described in Methods. The data are expressed as mean ± SD (n = 5).

The function of the chalaza is to support the developing seed with nutrients [42]. Therefore, we focused our further analysis on the seeds of the *arr22-2 *and *arr22-3 *mutants. However, we found no microscopically detectable difference in the size and morphology of the chalaza cells between the mutants and wild type. Moreover, embryonic development was not affected in *arr22-2 *and *arr22-3*, and the seeds also developed normally with respect to their size and mass (data not shown). We next investigated whether the loss of *ARR22 *function influences the metabolic state of the developing seeds. Siliques from different developmental stages were collected, homogenized and lyophilized. The mutant and wild type siliques exhibited the identical dry mass at each developmental stage tested (Fig. 6A and data not shown). The quantification of inorganic ions using HPLC chromatography, revealed no significant difference between wild type and the *arr22 *mutants at any developmental stage (Fig. 6B, C and data not shown). Similarly, there was no significant difference with respect to the carbohydrate and and amino acid content (Fig. 7 and data not shown). In conclusion, the loss of *ARR22 *function in the chalaza has no major detectable impact on silique and seed development and the seeds' nutrient content and composition.

### Complementation of *arr22 loss of function *mutants results in dwarf and sterile plants

**Figure 8:**
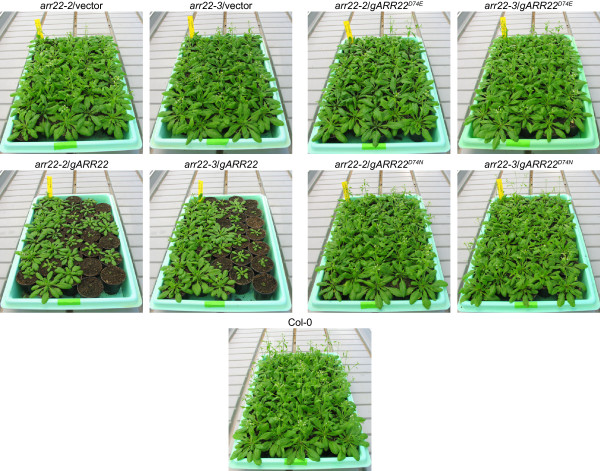
**Complementation of *arr22-2 *and *arr22-3 *lines with a wild type genomic *ARR22 *fragment results in a population of plants with a dwarf phenotype of different penetrance**. Images of 30-day-old *arr22-2 *and *arr22-3 *plants complemented with the wild type genomic *ARR22 *fragment (*gARR22*) or genomic fragments, in which the phosphorylatable Asp74 (D74) was mutated either to a Glu (*gARR22*^*D74E*^) or an Asn (*gARR22*^*D74N*^) are shown.

**Figure 9:**
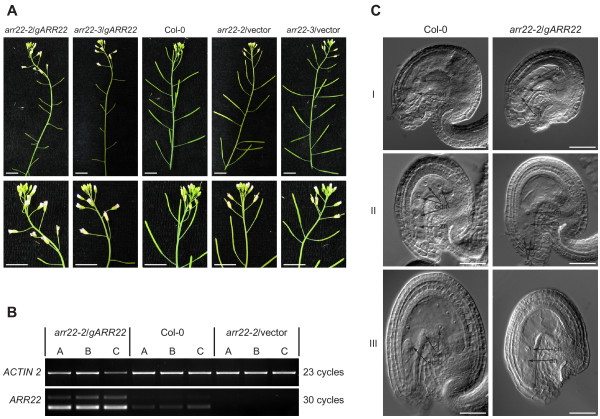
***arr22/gARR22 *plants show increased *ARR22 *transcript levels in the siliques but no alterations in female gametophyte development**. (A) Inflorescence images of *arr22-2 *and *arr22-3 *plants complemented with the wild type ARR22 genomic fragment (*gARR22*) or the empty vector (vector) (T1 generation). Details of the apical inflorescence are shown in the second row. The bars represent 10 mm. (B) *ARR22 *transcript levels in wild type and *arr22-2 *plants complemented with a wild type genomic *ARR22 *fragment (*gARR22*). Total RNA was isolated from the emerging siliques of the inflorescences in three replicas (A-C) and subjected to semiquantitative RT-PCR. PCR was performed with *ACTIN2 *or *ARR22 *specific primers at indicated numbers of cycles. (C) Differential interference contrast microscopy images of developing ovules and seeds of a sterile *arr22-2*/*gARR22 *plant and wild type. Ovules and seeds were excised from the siliques harvested from the same nod relative to the apex of the inflorescence. I, developed female gametophyte; II, fertilized seed with the zygote and four endosperm nuclei; III, early embryonic stage. The positions of the synergid nucleus (sn), egg cell nucleus (en), central cell nucleus (cn), zygotic nucleus (zn), endosperm nucleus (edn) and embryo (emb) are indicated by black arrows. The bars represent 50 μm. Figure legend text.

To genetically complement the insertion mutations, we introduced a genomic *ARR22 *DNA fragment into the *arr22-2 *and *arr22-3 *mutant lines. The *ARR22 *genomic fragment extended from 2003 bp upstream of the ATG start codon to 643 bp downstream of the stop codon. The T1 population of more than 200 *arr22-2 *and *arr22-3 *transformants carrying the *ARR22 *genomic construct (*gARR22*) showed a broad phenotypic variation in contrast to the plants transformed with the empty vector (Fig. 8). 22% of these transformants were dwarfed plants (category I), 35% had a reduced rosette size (category II) and 43% showed a normal rosette size (category III). All category I and category II plants were sterile. 78% of category III plants were sterile and 5% showed wild type fertility. 17% of category III plants had reduced fertility ranging from a few seeds to a few siliques *per *plant. The growth of the siliques in these plants was arrested at an early stage of development, and the abscission of the floral organs was delayed (Fig. 9A). A similar phenotype distribution was observed when the *gARR22 *construct was transformed into wild type *Arabidopsis *plants (data not shown).

Semi-quantitative RT-PCR using total RNA fom the dwarf and sterile *arr22*/*gARR22 *plants revealed that there was an over-accumulation of *ARR22 *transcript in the apical inflorescence compared to wild type (Fig. 9B). We, subsequently, compared the seed development in *arr22*/*gARR22 *transgenic and wild type plants using differential interference contrast microscopy. As shown in Figure 9C, the female gametophyte developed phenotypic identically in the wild type and *arr22*/*gARR22 *transgenic plants. In contrast to the wild type, the fully developed female gametophyte persisted longer in the *arr22*/*gARR22 *transgenic plants, the central nucleus did not divide and both synergids degenerated at the time when the floral organs were abscissed (Fig. 9C). However, the few seeds, which were occasionally produced in the siliques of *arr22*/*gARR22 *plants, were normal. In conclusion, our data suggest that a block of the fertilization, rather than a defect in early embryonic development, is the reason for *arr22*/*gARR22 *plants' sterility.

The observed fertilization block was not due to a difference in pollen tube attraction because wild type and *arr22*/*gARR22 *transgenic plants attracted GUS-stained wild type pollen tubes to an identical extent (data not shown). Reciprocal crossing experiments, however, revealed that the fertilisation of emasculated wild type and *arr22*/*gARR22 *plants using mature anthers from *arr22*/*gARR22 *plants was not successful (Table 1). The reason for this infertility was the anthers' physical resistance and reduced ability to open and to release the pollen rather than the sterility of the pollen (data not shown). As *ARR22 *is normally not expressed in anthers [36], the observed phenotype does not provide information with respect to its native function.

**Table 1:** Reciprocal crosses of complemented *arr22 *mutants with wild type

	developing siliques after 10 crosses [%]	
	
**plant**	**♂ Col-0**	**♀ Col-0**
*arr22-2*/vector	70	80
*arr22-3*/vector	100	80
*arr22-2/gARR22*	100	**0**
*arr22-3/gARR22*	80	**0**
*arr22-2/gARR22*^*D74E*^	90	70
*arr22-3/gARR22*^*D74E*^	70	80
*arr22-2/gARR22*^*D74N*^	80	100
*arr22-3/gARR22*^*D74N*^	80	100
Col-0	80	90

To address the question, whether ARR22 functions as a canonical response regulator or a histidine phosphatase *in planta*, we generated transgenic *arr22 *lines expressing ARR22 variants, in which the phosphorylatable Asp74 was mutated either to Glu or Asn. A Glu mutation mimicks a phosphorylated Asp and generates a dominant-active variant of a response regulator, whereas an Asp-to-Asn mutation mimicks a non-phopshorylated version, which acts dominant-negatively. The more than 200 independent *arr22 *plants carrying either the *gARR22*^*D74E *^or *gARR22*^*D74N *^construct did not show any developmental abnormalities but displayed the *arr22 *phenotype (Table 1; Fig. 8). These findings indicate that ARR22 does not act as a canonical response regulator *in planta*.

## Discussion

### *ARR22 *gene expression and intracellular localisation of ARR22

By using a *P*_*ARR22*_*::GFP *construct in combination with real-time RT-PCR and GFP immunolabelling, we located *ARR22 *gene activity to the chalaza cells of developing *Arabidopsis *seeds. This pattern is in general agreement with observation of Gattolin and colleagues [33], who also reported *ARR22 *expression in the funiculus of developing seed using a *P*_*ARR22*_*::GUS *reporter line. This difference in localisation could be due to a diffusion of the indigo stain out of the chalaza into the funiculus which we avoided in our transgenic line by using the GFP marker. Our intracellular localisation studies revealed that 35S promoter-expressed ARR22:GFP is predominantly but not exclusively located in the cytoplasm of plant cells. The mutation of the phosphorylatable Asp74 to Glu or Asn had no effect on this pattern suggesting that the phosphorylation of ARR22 does not influence its intracellular distribution.

### ARR22 interacts with a subset of AHPs in yeast and plant cells

Recently, Kiba and colleagues [30] reported that ARR22 has strong phospho-histidine phosphatase activity on phosphorylated AHP5 *in vitro*. Here we addressed the question whether ARR22 may target AHP5 only or whether it also may interact with other *Arabidopsis *TCS elements. Yeast two-hybrid data showed that ARR22 specifically interacts with AHP2, AHP3 and AHP5 but not with AHP1, AHP4 and AHP6 and the His kinase domains of AHK2, 3, 4 and ERS1. As indicated by the quantitative data, the interaction appears to be of low affinity and/or high kinetical dynamics. The *in planta *BiFC data substantiated the specific interaction of ARR22 with AHP2, AHP3 and AHP5 and suggest that ARR22 exerts its phospho-histidine phosphatase activity not only on AHP5 [30] but probably also on AHP2 and AHP3. AHP2, AHP3 and AHP5 are expressed in siliques and developing seeds [36,43] and are reported to be particularly important for *Arabidopsis *seed development [7].

### *arr22 *mutants show no morphological, physiological, developmental and metabolic defects

We identified two novel *arr22 *mutant alleles and proved the absence of the *ARR22 *transcript in their siliques. In comparison to the wild type, we could not detect any difference in the number, size and morphology of the chalaza cells during embryo development and we found no difference in seed size and silique mass in the mutants. A detailed metabolic analysis of ions, amino acids and carbohydrate content in the seeds also revealed no significant difference between *arr22 *mutant and wild type at any stage of seed development. These data show that ARR22 is not involved in these processes. In the view of the extremely restricted *ARR22 *expression in the chalaza and its phospho-histidine phosphatase activity, the lack of any detectable morphological and metabolic phenotype in the seeds of the *arr22 *mutants is surprising. A functional replacement of *ARR22 *by its paralog, *ARR24*, in the chalaza is unlikely because *ARR24 *gene activity is only observed in pollen and an *arr22/arr24 *double mutant also showed no phenotypic defects [33].

### Genomic complementation of the *arr22 *mutants induces pleiotropic phenotypes

The complementation of the *arr22-2 *and *arr22-3 *mutants with a genomic *ARR22 *fragment (*gARR22*) resulted in T1 transformants with severe developmental abnormalities, such as dwarfism, delayed abscission of flower organs and a pollen release defect resulting in sterility. The inability of the *arr22/gARR22 *anthers to release their pollen suggests a defect in the differentiation of the functional endothecium which is responsible for the rupture of the pollen sacs. The appearance of this pleiotropic phenotype depended on the presence of the phosphorylatable Asp74 [30] in *gARR22 *and was not observed in *arr22-2*/*gARR22*^*D74N *^and *arr22-3*/*gARR22*^*D74E *^transgenic plants. This finding indicates that both the gain-of-function (ARR22^D74E^) and the loss-of-function mutants (ARR22^D74N^) of the response regulator have lost their biological activity. If ARR22 functions as a canonical phospho-regulated response regulator like the A-type and B-type ARRs, we would have expected to identify plants within the *arr22*/*gARR22*^*D74E *^T1 transformant population showing a pleiotropic phenotype comparable to that of the *arr22*/*gARR22 *T1 transformants. The absence of such a phenotype in the *arr22*/*gARR22*^*D74E *^plants suggests that ARR22 acts as a phospho-histidine phosphatase exclusively. This genetic finding is in accordance with the biochemical evidence that although ARR22 is able to remove the phosphoryl residue from AHP5, it does not use it for autophosphorylation [30].

**Figure 10:**
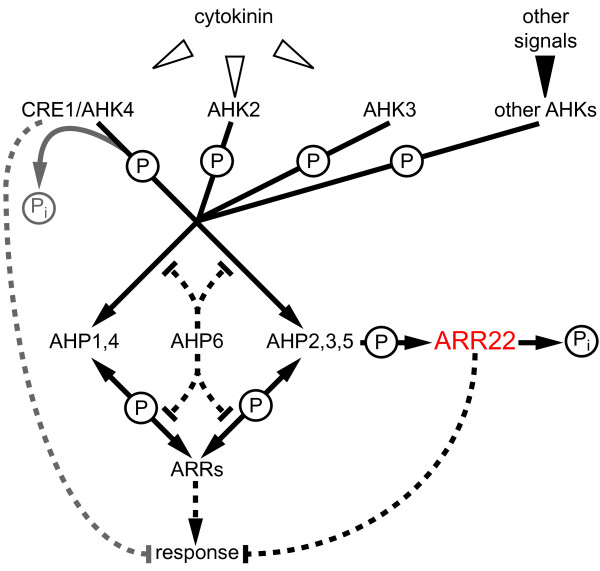
**Model of ARR22 action within the two-component signalling network in transgenic *Arabidopsis *plants**. Activation of the cytokinin receptors (AHK2 to 4) by cytokinin or other AHKs by their specific signal initiates a phosphorelay (drawn lines), which converges at the canonical AHPs (AHP1 to AHP5). Subsequently, the phosphoryl residues (P) are relayed to the A-type and B-type response regulator (ARRs), which are activiated by this phosphorylation and initiate the cellular responses (broken lines with arrow heads). Phosphorelay reactions catalyzed by receiver domains enable the phosphate flow in both directions. The phosphohistidine phosphatase ARR22 efficiently de-phosphorylates AHP2, 3 and 5, releases phosphate (P_i_) and, therefore, acts a "sink" for phosphoryl residues. Especially in tissues, in which ARR22 is normally not expressed, this "sink" action strongly disturbs the phosphoload of the TCS network and interferes with TCS-regulated developmental processes (broken lines with vertical end line) causing dramatic phenotypic abnormalities. A similar "sink" function is reported for AHK4/CRE1 in the absence of cytokinin [47]. The pseudophosphotransfer protein AHP6 lacks the conserved His residue essential for phosphorelays and is proposed to inhibit the phosphate flow within the TCS network by competing with other AHPs for interactions with AHKs and ARRs [25].

The pleiotropic *arr22*/*gARR22 *phenotype is reminiscent to that of *wol *allele in *AHK4/CRE1 *gene [44,45], *ahk2/ahk3/ahk4 *triple cytokinin receptor mutants [10,46], some higher order *ahp *[7] and B-type *arr *mutants [26]. From these data we suggest that even small traces of ARR22 in tissues, which normally do not contain the response regulator, may interfere with the hormone homeostasis and especially the cytokinin response pathway (Fig. 10). Mis-expression of ARR22 appears to have a particular negative effect on vegetative growth and possibly on the differentiation of connecting tissues such as the endothecium of the anthers. The observed dramatic developmental defects can be explained by the action of ARR22 as a very efficient AHP phosphatase and, therefore, as a strong phosphate "sink", which disturbs the two-component phosphorelay network in the extrinsic tissues (Fig. 10). Similarly, the enhanced phosphatase activity of the AHK4 receiver domain in the *wol *mutant is responsible for the *wol *phenotype [47] (Fig. 10).

An ectopic mis-expression of *ARR22 *in the frame of a genomic fragment can occur when the transgene is inserted in transcriptionally active, more-or-less open chromatin areas, especially in 5'-promoter regions – a process which is favoured by the selection of the transgenic plants [48,49]. This suggests that the activity of the *ARR22 *locus might be under the tight control of chromatin condensation mechanisms, which restrict the expression of the response regulator to the chalaza. A similar regulation of gene activity is known for many tightly controlled developmental transformations during the plant's life cycle [50-52].

## Conclusion

In our study, we provide evidence that ARR22 is a predominantly cytoplasm-localised and strictly chalaza-expressed response regulator. Furthermore, we substantiate by protein-interaction and genetic experiments earlier biochemical findings that ARR22 has phospho-histidine phosphatase activity and functions on phosphorylated AHP5 and probably also on AHP2 and AHP3 *in vivo*. However, due to the absence of any chalaza- or seed-related aberrant phenotype in the *arr22 *mutants, the true function of ARR22 remains currently obscure. Because the slightest mis-expression in non-chalaza tissues produces "artificial" phenotypes, the use of genomic *ARR22 *fragments or binary transactivation systems, such as the *pOp/LhG4 *or *AlcR/AlcA *system [53,54], for functional analyses of ARR22 is problematic. Therefore, the targeted replacement of the endogenous *ARR22 *gene by an (inducible) overexpression construct *via *homologues recombination is probably the only possible way to determine the *in planta *function. However, under the control of a tissue-specific and/or inducible promoter, the phospho-histidine phosphatase ARR22 could be a useful tool for studying AHP2, 3 and 5-dependent TCS processes in plants.

## Methods

### Plant growth conditions, isolation of *ARR22 *T-DNA mutant alleles, RT-PCR and qRT-PCR

Tobacco (*Nicotiana benthamiana*) and *Arabidopsis thaliana *(Col-0) plants were cultivated in the greenhouse (tobacco, temperature: day 25°C/night 19°C, humidity 60%, photoperiod: 14 h; *Arabidopsis*, temperature: 21°C, night 18°C, humidity 45%, photoperiod: 14 h).

T-DNA mutants were isolated according to Ríos and colleagues [41] from the collection at Max-Planck Institute in Cologne. The insertion alleles *arr22-2 *and *arr22-3 *were identified with a T-DNA left border primer (5'-CTACACTGAATTGGTAGCTCAAACTGTC-3') and *ARR22 *specific primers (forward: 5'-CCTCGTTCTATACTAGCAGATGGGTTCGAT-3', reverse: 5'-GACTTGCATGATTTTACCTCGGACGATAAG-3') PCR products containing the T-DNA/gene junctions were cloned into pCR4-TOPO vector (Invitrogen) and sequenced.

RNA for RT-PCR was isolated using RNaqueous^®^Kit (Ambion) and traces of genomic DNA were removed by TURBO DNA-free™ (Ambion). Subsequently, 1.5 μg of total RNA were reverse transcribed using oligo-dT primer with RevertAid™ H Minus M-MuLV Reverse Transcriptase (Fermentas) and resulting cDNA was used as a template for PCR with HotStart Taq polymerase (Genaxxon). PCR products were separated *via *agarose gel electrophoresis after different number of cycles for comparison with *ACTIN2 *at non-saturating conditions [55]. The sequences of the RT-PCR primers were as follows: ACT2 detF 5'-CTGCTCAATCTCATCTTCTTCC; ACT2 detR 5'-GACCTGCCTCATCATACTCG; ARR22 detF 5'-AACGATCGGAGGAATTTCTCAGACT-3'; ARR22 detR 5'-CGCTCTTCTTCTTGGTCAGCTACTGA.

For quantification of *ARR22 *transcript, RNA and cDNA was prepared as described above with addition of random primers for reverse transcription and used as a template for qRT-PCR with Platinum Quantitative PCR SuperMix-UDG (Invitrogen) in three replicas. TaqMan^®^Probes in combination with ABI PRISM 7700 (Applied Biosystems) detection system according to manufacturer recommended protocol were used to monitor the amplification. To normalize the expression data, 18S rRNA was quantified using Eukaryotic 18S rRNA Endogenous Control (VIC/TAMRA Probe) kit (Applied Biosystems) and the amplifacation efficiency of *ARR22 *and 18S rRNA was proved to correspond. Sequences of *ARR22 *oligonucleotides could be sent upon request.

### Cloning strategy, Entry clones and site-directed mutagenesis

All clones used in our experiments were constructed using Gateway™ technology (Invitrogen). The Entry clones were either obtained via BP-reaction in pDONR201 or pDONR207 or through TOPO-reaction using the pENTR/D-TOPO vector (CTR1, AHK5; all vectors Invitrogen). The templates used to clone all genes were cDNA preparations derived from *Arabidopsis *roots, leaves or siliques. To avoid spontaneous mutations, the Entry clones of AHK2, AHK3, AHK4 and AHK5 were cloned and propagated in the *E. coli *strain CopyCutter™ (Epicentre). The reverse primers contained no stop codon to enable C-terminal fusions or a stop codon was included for N-terminal fusions. Sequences of forward and reverse primers could be sent upon request. Construction of clones containing only histidine kinase domains was performed using the full-length Entry clones of the AHKs as templates and following primers: AHK2^HK ^(aa562-873) – 5'-*attB1*-TAATGAACCGAATTGCGACAGTTGAAGAG and 5'-*attB2*-TTACGACGTATTTGTTTCTGCTTTCC; AHK3^HK ^(aa425-728) – 5'-*attB1*-TAATGAGTCGAATACACAAAGTTGAAGAA and 5'-*attB2*-TTAAGCTGGTTGCATCCCATTGGAAAATAC; AHK4^HK ^(aa424-743) – 5'-*attB1*-TAATGCACATAGTAAAAGTCGAAGATGAT and 5'-*attB2*-TTACGCACTGCATTTATCGCATTTCTCTAA; ERS1^HK ^(aa317-613) – 5'-*attB1*-TAATGCACGCTCGTGACCAGCTTATG and 5'-*attB2*-TCACCAGTTCCACGGTCTGGTTTGTGA. Site-directed mutagenesis of ARR22 was carried out on the *ARR22 *Entry clones using QuikChange^® ^Site-Directed Mutagenesis Kit (Stratagene) and a pair of fully complementary primers for the designed region GGCGAAGCTAGCTTCGACCTTATTCTAATGGAGAAGG containing D74E mutation and *Nhe*I restriction site or CGACCTTATTCTCATGAATAAGGAAATGCCTGAG containing D74N mutation and *Pag*I restriction site.

### *Agrobacterium *infiltration of *Nicotiana benthamiana *leaves, BiFC protein-protein interactions and confocal microscopy

The binary vectors for expression of the GFP/RFP fusion proteins under the control of 35S promoter were constructed via LR-reaction using the corresponding Entry clones and destination vectors pH7FWG2.0, pH7WGF2.0 and pB7RWG2.0 [56]. The p19 protein from tomato bushy stunt virus cloned in pBIN61 [57] was used to suppress gene silencing. All vectors were transformed in *Agrobacterium tumefaciens *strain GV3101 pMP90 and prior infiltration resuspended in AS-medium (10 mM MgCl2, 150 μM acetosyringone and 10 mM MES pH 5.7) to OD_600 _0.8 according to Grefen and colleagues [58] Abaxial epidermis of infiltrated tobacco leaves was assayed for fluorescence by CLSM 2–3 days post infiltration. For BiFC the *AHP *cDNAs were recombined *via *LR-reaction into pSPYNE-35S and *ARR22 *into pSPYCE-35S [40] and infiltrated to the tobacco leaves as described elsewhere [58]. The expression of the BiFC fusions was determined by SDS-PAGE and western blot using the tissue of transfected leaves according to Walter and colleagues [40].

### Protein-protein interactions in yeast two-hybrid system

Yeast two-hybrid experiments were performed using the Matchmaker™System (Clontech). Plasmids were constructed by LR-reaction of corresponding Entry clones and destination vectors pGBKT7-DEST or pGADT7-DEST. Yeast strain PJ69-4A was transformed using lithium acetate/SS-DNA/PEG method [59]. After 3 days of growth on vector selective media (CSM-L^-^, W^-^), 5 independent clones were pooled and propagated to an OD_600 _1.0 in liquid, vector-selective media. Subsequently, 7.5 μl of culture were dropped on vector- and interaction-selective media (CSM-L^-^, W^-^, Ade^-^) and incubated at 28 C. At day 2 the growth of the clones was monitored. In addition, liquid yeast cultures were harvested and analyzed by western-blot to determine the correct expression of the fusion proteins [59]. Activity of β-galactosidase activity was measured using 3 independent yeast clones *per *transformation. 50 ml yeast culture was grown in vector-selective media to OD_600 _2.0, harvested and lysed using freeze-thaw method and vortexing with glass beads in 700 μl of Z-buffer (60 mM Na_2_HPO_4_, 40 mM NaH_2_PO_4_, 10 mM KCl, 1 mM MgSO_4_, pH 7.0). The total protein amount of the lysate was quantified by Bradford protein assay and β-galactosidase activity measured according to Grefen and colleagues [59].

### Generation and analysis of *ARR22 *promoter:GFP line and GFP immunolabelling

*ARR22 *promoter DNA fragment was amplified from *A. thaliana *genomic DNA using Phusion™ polymerase (Finnzymes) and the primers ARR22pF 5'-*attB1*-GCAGCAGATGACTTAACTCTCCA, ARR22pR 5'-*attB2*-GGGTACCTCCGGTGGATTTTGT. The promoter fragment was cloned into pDONR207 *via *BP-reaction and verified by sequencing. The fragment comprises the region 2003 bp upstream of the start codon including the first 27 bp of the *ARR22 *coding sequence. The fragment was translationally fused to the *GFP *reporter gene via LR-reaction in the destination vector pMDC107 [60], and the construct transformed in the *Agrobacterium tumefaciens *strain GV3101 pMP90. *Arabidopsis *plants were transformed using the flower-dipping method. For selection of transgenic plants, seeds were vapor sterilized as described in Grefen *et al *[58] and T1 seedlings selected for hygromycin resistance in a growth chamber at 21°C under long-day conditions for 14 days on 0.5× Murashige-Skoog medium supplemented with 1% (w/v) sucrose, 0.8% (w/v) phytoagar and 25 μg/ml hygromycin B. Resistant plants were propagated on soil and siliques from several independent transgenic lines (T2) were tested for fluorescence by CLSM.

For GFP imunolabelling, seeds were fixed for 90 min in 4% (v/v) formaldehyde in MTSB, pH 7.0, followed by a treatment with 8% (v/v) formaldehyde in MTSB for additional 90 min, then infiltrated with a mixture of sucrose and polyvinyl pyrrolidone [61] and frozen in liquid nitrogen. 300–500 nm cryo-sections were cut at -80°C, mounted on coverslips, labelled with a rabbit anti-GFP antibody (diluted 1:250 in blocking buffer; Abcam) and a goat anti-rabbit antibody coupled to Cy3 (diluted 1:400 in blocking buffer; Dianova) for 1 h, each. Nuclei were stained with DAPI. Cryo-sections were embedded in Moviol for microscopy. In the control, the rabbit anti-GFP antibody was omitted. Labeled cryo-sections were viewed with a Zeiss Axiophot light microscope (63×/1.4 oil immersion objective).

### HPLC analysis of *arr22 *mutant siliques

For sample preparation as well as for analytics Milli-Q water and chemicals in highest purity were used exclusively. The siliques were collected in five replicas for each line at the developmental stage, where the first senescing siliques appeared on the inflorescences, frozen in liquid nitrogen, homogenized and lyophilized. To 50 mg lyophilized sample 750 μl of a chloroform/methanol/water [5/15/3 (v/v/v)] mixture were added, mixed and afterwards centrifuged for 5 minutes at 14.000 rpm and 4°C. 400 μl of the supernatant were transferred into a new vial and pooled with another 400 μl obtained by similar treatment of a second replicate of the same sample. To the 800 μl pooled supernatant 8 μl of a 2.5 mM norleucin solution were added as internal standard for the amino acid analysis. After thorough mixing 376 μl water and 250 μl chloroform were added, mixed and afterwards centrifuged as described before. 900 μl supernant were collected without touching the interface, evaporated to dryness and redissolved in a mixture of 400 μl water and 600 μl acetonitrile. Undissolved residues were removed by centrifugation (30 seconds,14.000 rpm, 4°C). The supernatant was transferred into a new vial and again evaporated to dryness. The pellet was redissolved in 400 μl water. Further workup of the sample was done by solid phase extraction (SPE) with Sep-Pac C_18 _columns (Waters). For this SPE columns were washed with 1 ml acetonitrile followed by 1 ml water for column equilibration. Then the sample was loaded onto the SPE column and eluted with 500 μl water. Vacuum was used to collect the entire liquid of the column. Resulting 900 μl eluate were evaporated to dryness afterwards. For amino acid analysis the resulting pellet was dissolved in 100 μl lithium diluent. For sugar and ion analytics pellets were dissolved in 100 μl water. If necessary, storage of samples was done at – 20°C. Before analysis samples were filtered through a 0.5 μm PVDF filter. For amino acid analysis ninhydrin post column derivatization technology was used. The modular HPLC system consisted of two high pressure pumps, autosampler, and UV/Vis – detector from Kontron/BioTek. For post column derivatization Pickering Laboratories ninhydrin pump, column oven and reactor were used. UV/Vis detection of the amino acid derivatives was done at 440 and 570 nm. For amino acid separation the lithium cation exchange column from Pickering Laboratories (4 × 100 mm, particle size: 5 μm) was used with the following ternary gradient at a flow rate of 340 μl/min. 0 min: 100% A (96.0% H_2_O, 0.7% lithium citrate, 0.6% lithium chloride, pH 2.8) 12 min: 100% A 48 min: 35% B (97% H_2_O, 0.9% Lithium citrate, 2.0% lithium chloride, pH 7.5) 90 min: 100% B 95 min: 100% B 120 min: 94% B and 6% C (99% H_2_O, 0.6% lithium chloride, 0.4% lithium hydroxide, pH 11.8) 150 min: 94% B and 6% C. For Ion analysis a Dionex DX120 system equipped with suppressed conductivity detection was used in isocratic mode. For cation separation an Ion Pac-CS12A column (4 × 250 mm) was eluted with 20 mM methansulfonic acid at a flow rate of 1 ml/min. Anions were separated on an Ion Pac AS 9-HC column (4 × 250 mm) with 9 mM sodium carbonate as mobile phase at a flow rate of 1 ml/min. For carbohydrate analysis a Kontron/BioTek HPLC system equipped with an electrochemical detector (Bischoff) was used for high pH anion exchange chromatography (HPAEC). Isocratic separation was done on a CarboPac PA1 column (4 × 250 mm) from Dionex. For elution a 30 mM sodium hydroxide solution was used at a flow rate of 0.80 ml/min.

### Complementation of *arr22-2 *and *arr22-3 *mutants

A DNA fragment containing the *ARR22 *gene was amplified from *Arabidopsis *genomic DNA using Phusion™ polymerase (Finnzymes). The forward primer used previously for the cloning of the *ARR22 *promoter was combined with the reverse primer ARR22gR (5'-*attB2*-CCCTATCTTCTTAATGACATATAGTATTGG-3'), and the obtained DNA fragment comprising the region 2003 bp upstream of the ATG start codon and 643 bp downstream of the stop codon was cloned into pDONR207 *via *BP-reaction and verified by sequencing. Site-directed mutagenesis of Asp74 was carried out on the Entry clone as described above. All DNA fragments were recombined *via *LR-reaction into destination vector pMDC123 [60]. The *arr22-2 *and *arr22-3 *lines were transformed as described above and transgenic seedlings selected on soil by spraying with 0.05% (v/v) BASTA. Transgenic T1 plants were sorted to different phenotypic categories, photographed and siliques or upper parts of inflorescences were collected and tested for the *ARR22 *transcript amount by RT-PCR (see above). For microscopy of developing seeds, the inflorescences were submerged in ethanol:acetic acid (3:1) fixation solution for several hours. Subsequently, the tissue was rehydrated for 30 min in a series of 70%, 50% and 20% (v/v) ethanol followed by 20 min incubation in a clearing solution consisting of chloral hydrate : water : glycerol mixture (8:3:1 w/w). Seeds were excised from the siliques and mounted on slides in clearing solution for differential interference contrast microscopy.

### Microscope image acquisition

CLSM was performed using a Leica TCS SP2 confocal microscope (Leica Microsystems GmbH). All CLSM images were obtained using Leica Confocal Software and the HCX APO LW 20×/0.5 or the HCX PL APO 63×/1.2 W water-immersion objective. GFP and RFP channels were acquired by simultaneous scanning using 488-/568-nm laser lines for excitation. The signals were detected between 500–530 nm for GFP and 590–630 nm for RFP. YFP signal was detected between 550 and 580 nm after excitation by 488 nm laser. Microscopy of developing seeds was carried out using a Nikon Eclipse 90i microscope equipped with a 40×/0.75 objective and a CCD camera applying differential interference contrast. The images were acquired using MetaMorph software (Molecular Devices) and processed using Adobe Photoshop 9.0.

## Authors' contributions

JH performed most of the cloning, the molecular and cell biological expression studies, the intracellular distribution and BiFC experiments, the detailed molecular characterization of the *arr22 *T-DNA insertion mutants and generated and characterized the *arr22*/*gARR22 *transgenic lines. Furthermore, JH prepared the samples for the HPLC analyses, prepared the figures and helped to draw the manuscript. CG cloned the yeast two-hybrid constructs, performed the yeast two-hybrid analysis and intensively discussed with JH the experiments. KWB carried out the initial identification of the *arr22 *mutants in the Cologne collection. AH constructed the Gateway-compatible yeast two-hybrid vectors. Y–DS did the immunolabelling experiments. BS and MS performed the HPLC analyses. CK provided the Cologne seed collection and helped to draft the manuscript. KH conceived and coordinated study and drafted the manuscript. All authors read and approved the final manuscript.
